# A Correlation Study of the Microbiota Between Oral Cavity and Tonsils in Children With Tonsillar Hypertrophy

**DOI:** 10.3389/fcimb.2021.724142

**Published:** 2022-01-28

**Authors:** He Xu, Bijun Tian, Weihua Shi, Jing Tian, Xuexi Zhang, Jin Zeng, Man Qin

**Affiliations:** ^1^ Pediatric Department, Peking University School and Hospital of Stomatology & National Center of Stomatology & National Clinical Research Center for Oral Diseases & National Engineering Research Center of Oral Biomaterials and Digital Medical Devices, Beijing, China; ^2^ Department of Otolaryngology, Head and Neck Surgery, Beijing Children’s Hospital, Capital Medical University, National Center for Children’s Health (NCCH), Beijing, China; ^3^ Department of Otorhinolaryngology - Head and Neck Surgery, Peking University Third Hospital, Beijing, China

**Keywords:** tonsillar hypertrophy, saliva, supragingival plaque, microbiota, Illumina sequencing, 16S rRNA gene, random forest model

## Abstract

Tonsillar hypertrophy is a common disease in 3-to-6-year-old children, which may cause serve symptoms like airway obstruction. Microbiological factors play an important role in the etiology of tonsillar hypertrophy. As the starting point of digestive and respiratory tracts, the microbial composition of the oral cavity is not only unique but also closely related to the resident microbiota in other body sites. Here we reported a correlation study of the microbiota between oral cavity and tonsils in children with tonsillar hypertrophy. Saliva, supragingival plaque, and wiped samples from the tonsil surface were collected from both tonsillar hypertrophy patients and participants with healthy tonsils and were then analyzed using Illumina Miseq Sequencing of the 16S rRNA gene. In the tonsillar hypertrophic state, more genera were detected on the tonsil surface than in the tonsil parenchyma, with more intra-microbiota correlations. When tonsillar hypertrophy occurred, both the oral cavity and tonsil surface endured microbiome shift with increased genera category and more active bacterial interactions. Over half of the newly detected genera from the tonsillar hypertrophic state were associated with infection and inflammation process or exhibited antibiotic-resistant characters. Of each individual, the microbial composition and structure of saliva seemed more similar to that of the tonsil surface, compared with the supragingival plaque. In salivary microbiota, genus *Johnsonella* might be relative with the healthy state of tonsils, while *Pseudoxanthomonas* might be relative with tonsillar hypertrophy. Our study supported the link between oral microbiota with the healthy and hypertrophic states of tonsils and may provide new directions for future researches in the specific role of oral microbiota in the etiology of tonsil diseases.

## Introduction

As the starting point of both digestive and respiratory tracts, the microbiota of the oral cavity is not only unique but also closely related with the resident microbiota in other body sites. Previous studies documented that the oral microbiota was closely associated with the etiology of multiple infectious diseases and systemic diseases, such as inflammatory bowel disease (Xun et al., 2018), bacterial endocarditis (Kuwabara et al., 2016), and diabetes (Riviere et al., 2002; Spahr et al., 2006; de Smit et al., 2011; Casarin et al., 2013; Han and Wang, 2013; Zhang et al., 2013). Generally, oral microorganisms can impact systemic health through approaches of metastatic infection, bacteremia or toxemia, or immune response (Li et al., 2000). Based on these, oral microbiota was proposed as a potential candidate to screen and monitor some of these particular diseases, such as in inflammatory bowel disease (Xun et al., 2018)

Palatine tonsils are a pair of oblate oval lymphoid organs that are located in the tonsil fossa, which are anatomically adjacent to the oral cavity. During swallowing, saliva passes through the pharynx and brings oral microorganisms over the surface of tonsils. Previous studies indicated certain correlations between oral microbiota and tonsil surface microbiota in both healthy and disease conditions. In healthy people, saliva and tonsil surface shared a majority of microbiota composition (Segata et al., 2012; Wang et al., 2018). Moreover, anaerobes on the tonsil surface were detected significantly increased when the third mandibular molar developed pericoronitis (Rajasuo et al., 1996); while the oral rinse microbiome composition was significantly altered in patients with tonsillitis (Yeoh et al., 2019).

Tonsillar hypertrophy is a common disease with a morbidity of 1%–3% in 2-to-6-year-old Chinese children (Gao Shuwei and Xu, 2020). Enlarged tonsils may cause airway obstruction, snoring, mouth breathing, and other symptoms, while prolonged mouth breathing may lead to oromandibular deformities (Mora et al., 2003). Therefore, the management of tonsillar hypertrophy is of great importance to children (Souza et al., 2013; Soylu et al., 2016). The common etiology of tonsillar hypertrophy is currently considered to be long-term inflammatory stimulation and is closely associated with microbiota (Xue et al., 2014). However, the exact pathogenesis of tonsillar hypertrophy has not been identified. Studies of tonsillar microbiota may provide deeper insight into the pathogenesis of tonsillar hypertrophy and may help screen out the pathogenetic bacteria. Nevertheless, as tonsils are located in the pharynx, it is scarcely possible to acquire uncontaminated tonsillar microbial samples from children during wakefulness due to the extreme discomfort during sample collection. On the contrary, collection of oral microbial samples is non-invasive and is much easier.

Therefore, in this study, we aimed to investigate the relationship between oral microbiota and tonsillar microbiota in both tonsillar healthy and tonsillar hypertrophy state, and to seek for bacteria that may be related with tonsillar health or hypertrophy. Also, we hope to gain more insight into the influence of oral microbiota on the body’s general situation.

## Materials and Methods

### Inclusion and Exclusion Criteria

Three-to-six-year-old inpatients with complete primary dentition were recruited from the surgery ward of the Department of Otolaryngology in Peking University Third Hospital and Beijing Children’s Hospital, Capital Medical University, from October 2018 to September 2020. The status of palatine tonsils was appraised by professional otolaryngologists. Participants in the tonsillar hypertrophy group (T group) were inpatients that were diagnosed as palatine tonsillar hypertrophy and had been scheduled for tonsillectomy under general anesthesia. Participants in the control group (H group) were inpatients with healthy tonsils and needed surgical treatment under general anesthesia because of other otolaryngological diseases expect for tonsillar hypertrophy.

The exclusion criteria were (1) history of acute tonsillitis; (2) history of antibiotic administration within the previous 1 month; (3) use of immunosuppressive agents; (4) presence of suspicious malignant lesions; (5) presence of residual crowns or roots in the oral cavity; (6) presence of abscess or fistula in the oral cavity; (7) oral diseases other than caries; and (8) eruption of permanent teeth.

### Clinical Examination and Sample Collection

Oral examination of all the participants was performed by one pediatric dentist under natural light in the otolaryngology ward. Consistency in oral examination and caries diagnosis for this specular dentist was ensured by training with two other attending dentists prior to the initiation of the study. The κ value for intra-examiner agreement in the diagnosis of caries was 0.89. Caries was recorded according to the 1997 WHO caries diagnosis criteria (WHO, 1997). A questionnaire about general condition and oral hygiene habits was filled out by the participant’s parents.

Participants were asked to refrain from toothbrushing for at least 12 h and fast for 2 h before the sample collection. Oral samples were collected by the same pediatric dentist prior to the otolaryngology surgery, including 2 ml non-irritating saliva and supragingival plaque from all sound smooth surfaces which were collected using a sterilized disposable micro-applicator. After sampling of plaque, the tip of the applicator was cut off using sterilized scissors and transferred into a sterile 1.5-ml centrifuge tube containing 1 ml TE buffer (50 mM Tris–HCl, 1 mM EDTA; pH = 8) (Xu et al., 2018).

The collection of tonsillar samples was performed by otolaryngologists. Cotton swabs were used to acquire biological film samples from the surface of tonsils when the patient was already under general anesthesia, but before intraoral rinsing and disinfection. During the whole process of sampling, the swab should not touch oral mucosa, pharyngeal cavity mucosa, or saliva. After collection, the front end of the swab was cut off with sterilized scissors and placed in a sterile 1.5-ml centrifuge tube containing 1 ml TE buffer. In the T group, when the tonsillectomy was done, the upper 1/3 of the removed tonsillar tissue was cut off using sterile tissue scissors and the internal tonsil tissue was obtained using a sterile surgical blade. The excised tonsil tissue was placed in a centrifuge tube with 10 ml of sterile saline. All the samples were placed in an ice box and placed in a -20°C environment for temporary storage within 2 h and transferred to a -80°C environment within 1 week (Jensen et al., 2013).

### Total Genomic DNA Extraction and Illumina Sequencing Analysis of 16S rRNA Gene Amplicons

The total bacterial DNA was extracted using the QIAamp^®^ DNA Micro Kit (Chen et al., 2016) from saliva, supragingival plaque, and tonsil surface swabs, while bacterial DNA extraction from the tonsil tissue was performed using the PowerSoil DNA Isolation Kit (Cheng et al., 2018).The quantity and quality of the extracted DNA were evaluated using a NanoDrop 8,000 spectrophotometer (Thermo Fisher Scientific, Waltham, MA, United States). The V3–V4 hypervariable region of the 16S rRNA gene was PCR amplified and then sequenced by an Illumina Miseq Sequencing platform (paired-end 300).

### Bioinformatics Analysis

Quality control of Fastq data was performed using Trimmomatic (v 0.36) and Pear (v 0.9.6). For Trimmomatic, a sliding window strategy was used with the window size set to 50 bp, the average quality value of 20, and the minimum retained sequence length of 120 bp (Zhang et al., 2014). Pear was used to remove sequences with N. According to the overlap relation between paired end (PE) reads, the sequences were merged using Pear and Flash (v 1.2.0) (Bolger et al., 2014). Chimera was removed using the UCHIME method based on GOLD database (Edgar et al., 2011). The operational taxonomic unit (OTU) was generated by the UPARSE pipeline (Edgar, 2013).

According to sampling sites and health status of tonsils, the samples were divided into seven subgroups: tonsil surface microbiota in the H group (H_T) and the T group (T_T), salivary microbiota in the H group (H_B) and the T group (T_B), supragingival plaque microbiota in the H group (H_P) and the T group (T_P), and the internal tonsil tissue microbiota in the T group (T_Th). The Kruskal–Wallis test (KW test) was used to compare the differences in questionnaire results between the T group and the H group, with *p* < 0.05 considered to be significantly different. Alpha and beta diversity analyses were performed using Quantitative Insights into Microbial Ecology (QIIME) (Caporaso et al., 2010). Alpha diversity was estimated using richness index (Chao1), phylogenetic diversity index (PD whole tree), evenness index (Pielou), and Shannon–Weiner index. Beta diversity was measured with the Bray–Curtis distance matrix. Non-parametric multivariate analysis of variance (PERMANOVA analysis) was performed using the vegan package in R studio (v 1.3.1093) for comparison of differences between subgroups, with *p*-value correction using the Bonferroni method. Taxonomies were assigned by using BLAST against eHOMD database (v 15.1) using the RDP classifier, with a confidence threshold of 0.7. Metastats analysis was used to compare the differences of relative abundances with *p* < 0.05 considered as significantly different. Spearman correlation was calculated using R studio software and visualized with Cytoscape (v 3.8.2). Spearman correlation coefficient |r| > 0.6 was considered as a strong correlation.

### Screening of Potential “Related Bacteria” in the Oral Microbiota for the Hypertrophic or Healthy Status of Tonsils

Random forest classification models were performed across subgroups of the tonsil surface, plaque, and salivary microbiota in order to search for “related bacteria” that were closely related with the healthy or hypertrophic status of tonsils. A 10-fold cross-validation with five replications was performed to determine the number of “related bacteria.” Six candidate genera from the tonsil surface microbiota were screened out as indicators for the health status of the tonsils. Then, these six genera were located and compared across subgroups of the saliva and supragingival plaque microbiome. Genera that met both the following two criteria were defined as the “related bacteria” for the hypertrophic or healthy status of tonsils in oral microbiota: (1) the changing trend of relative abundance of the bacteria between healthy and disease states in oral microbiota was the same as that in tonsil surface microbiota and (2) the importance index within the top 25% in the corresponding random forest model.

## Results

### Participants

A total of 17 children in the T group and 18 children in the H group were recruited initially in this study. Due to the limitations of research conditions, tonsillar samples were failed to obtain from 3 children in the T group and 6 children in the H group. Finally, 14 children in the T group and 12 children in the H group were recruited. There were 12 boys and 2 girls in the T group with a mean age of 4.7 years, and 6 boys and 6 girls in the H group with a mean age of 4.5 years. The KW test showed no significant difference in gender, age, the decayed, missing, or filled teeth (dmft) index, or the decayed, missing, or filled surface (dmfs) index (*p* > 0.05). Statistical analysis of the questionnaire showed no significant differences between groups in terms of maternal gestation, delivery, general health, family background, or oral hygiene practices (*p* > 0.05) (**Supplementary Table 1** and **2**).

### General Information From Sequencing

A total of 7,451,223 high-quality reads were generated from a total of 92 samples in this study. On average, 80,991 reads per sample were obtained for analysis; good coverage was 80.69%–99.29%. The high-quality reads were concentrated in the range of 400–440 bp in length. After OTU clustering, a total of 1,320 OTUs were generated. A mean of 244.94 OTUs were obtained per sample, with a minimum value of 84 OTUs and a maximum value of 420 OTUs. A total of 461, 485, and 494 OTUs were obtained in the H group and 740, 765, and 719 OTUs in the T group, respectively, in the microbiota of tonsil surface, saliva, and supragingival plaque. In addition, 469 OTUs were obtained in the internal tonsil tissue. Species annotation results showed that a total of 22 phyla, 55 classes, 89 orders, 155 families, and 312 genera were obtained.

Of all the samples in each subgroup, five phyla of Bacteroidetes, Proteobacteria, Fusobacteria, Firmicutes, and Actinobacteria constituted about 98.27%–99.19% of all the microbiota (**Supplementary Figure 1**). At the genus level, ten genera exhibited relatively high abundances in all the subgroups, including *Streptococcus*, *Neisseria*, *Prevotella_7*, *Veillonella*, *Gemella*, *Haemophilus*, *Porphyromonas*, *Prevotella*, *Fusobacterium*, and *Leptotrichia* (**Supplementary Table 3**).

### Variation of Oral and Tonsillar Microbiota From Tonsillar Healthy Status to Tonsillar Hypertrophic Status

**Figure 1:**
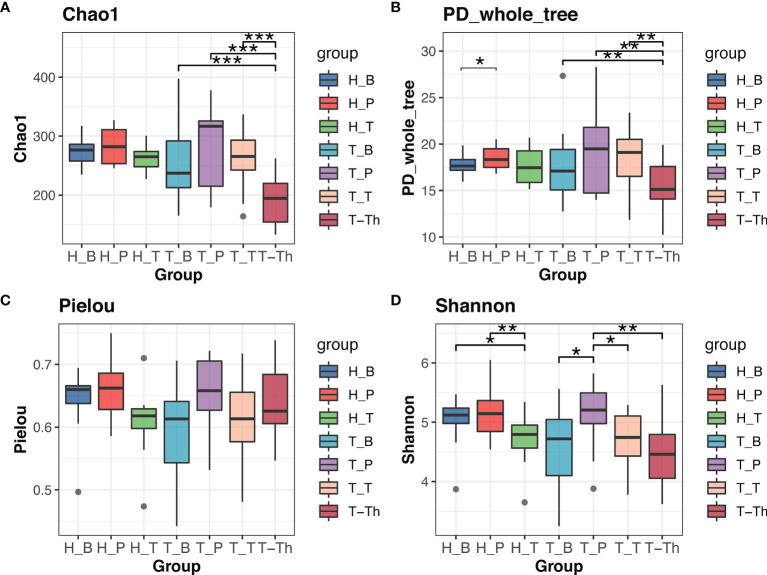
Alpha diversity comparison in Chao1 **(A)**, PD_whole_tree **(B)**, Pielou **(C),** and Shannon **(D)** indexes within all the subgroups. *p < 0.05, **p < 0.01, ***p < 0.001.

Comparison was made between the control group and tonsillar hypertrophy group to identify the variation of microbiome in saliva, supragingival plaque, and tonsil surface, respectively. No significant differences were found in alpha or beta diversity between the two health states (*p* > 0.05) (**Figure 1** and **Supplementary Table 4**).

At the phylum level, the relative abundance of Chloroflexi was significantly higher in tonsillar hypertrophic status both on tonsil surface (*p* < 0.01) and in supragingival plaque (*p* < 0.01), while the relative abundance of Verrucomicrobia was significantly higher on the tonsil surface (*p* < 0.01). No significant variation was detected at the phylum level in the salivary microbiota between the two different healthy statuses of tonsils (*p* > 0.05).

**Figure 2:**
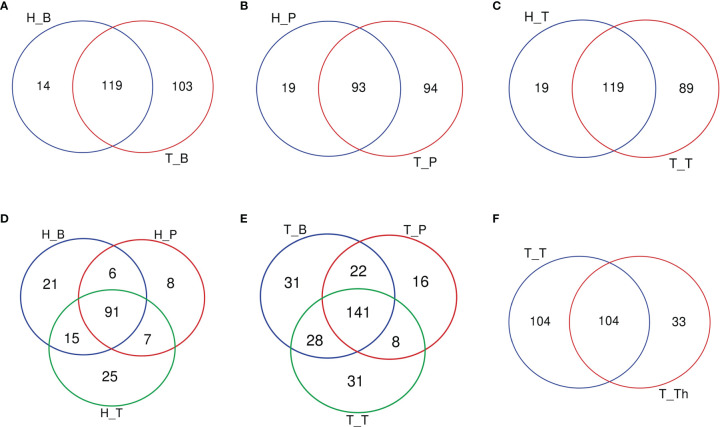
Venn diagrams of the number of genera common/unique within different subgroups. **(A)** Salivary microbiota within the H_B and T_B subgroups. **(B)** Supragingival plaque microbiota within the H_P and T_P subgroups. **(C)** Tonsil surface microbiota within the H_T and T_T subgroups. **(D)** Microbiota of three niches in tonsil healthy participants. **(E)** Microbiota of three niches in tonsillar hypertrophy patients. **(F)** Microbiota within tonsil surface and parenchyma in tonsillar hypertrophy patients, which were the T_T and T_Th subgroups.

**Figure 3:**
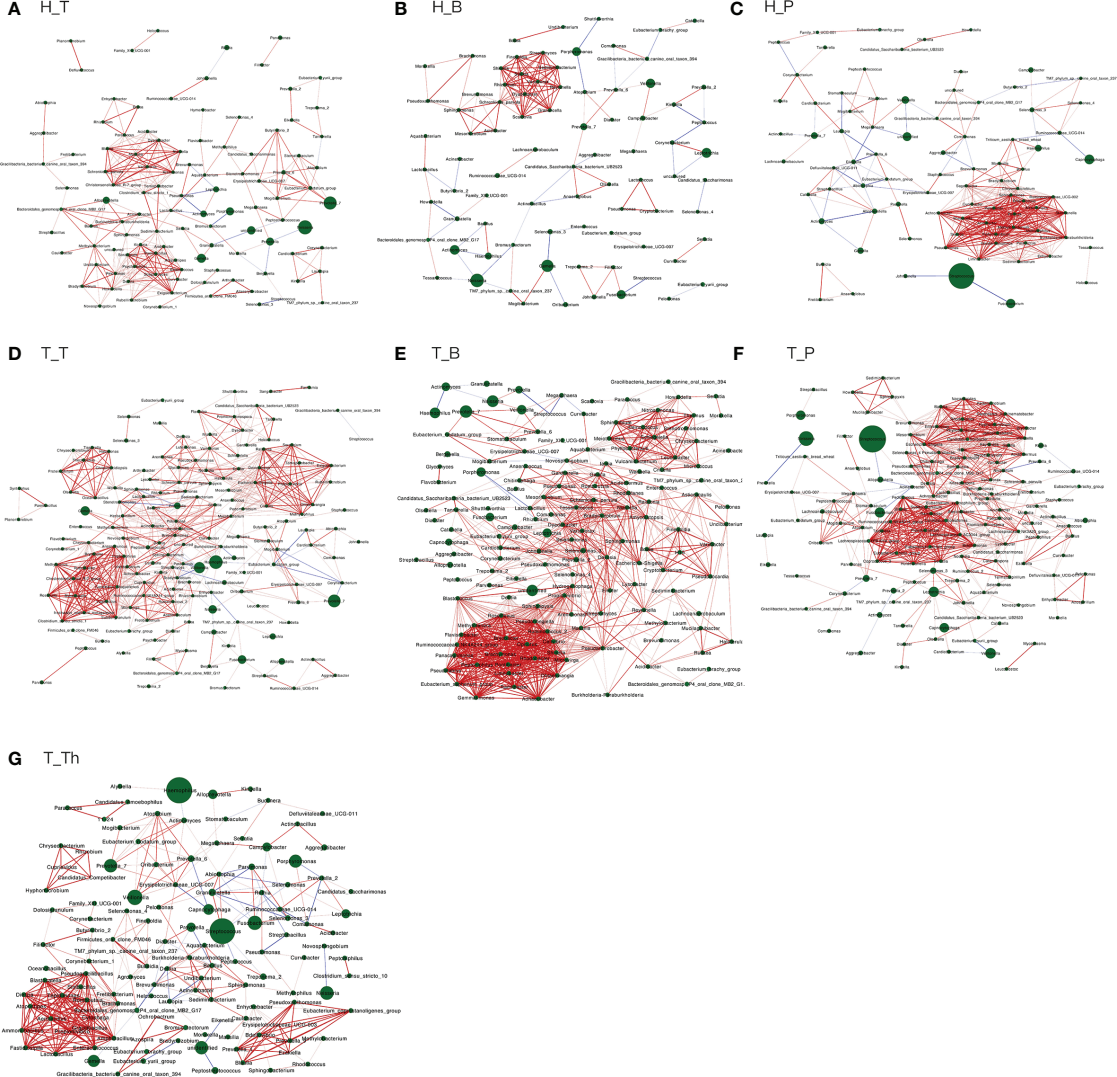
Co-occurring network and corresponding OTUs within each subgroup. **(A)** H_T, **(B)** H_B, **(C)** H_P, **(D)** T_T, **(E)** T_B, **(F)** T_P, and **(G)** T_Th. Correlations with Spearman correlation coefficient |r| > 0.6 and p < 0.01 are shown. Red and blue edges indicate positive and negative correlations. The width of each edge is proportional to the absolute value of Spearman’s correlation coefficients. The area of each node is proportional to the corresponding OUT’s relative abundance.

The number of genera increased in all the three niches from healthy to tonsillar hypertrophic state (**Figure 2** and **Supplementary Table 5**). The microbial composition in the tonsillar hypertrophic status included most of the genera that are detected in tonsillar healthy status; these shared genera constituted the majority of tonsil surface microbiota with relative abundance of 99.72%–99.87%; 89–103 additional genera were detected only in tonsillar hypertrophic status but at relatively low relative abundances (0.13%–0.28%). Among them, 30 genera were detected across all the three niches, including *Sphingopyxis*, *Nocardioides*, *Pseudonocardia*, *Lachnospiraceae NK3A20 group*, *Ruminococcus 2*, *Chitinophaga*, *Gaiella*, *Peptoniphilus*, *RB41*, *Ramlibacter*, *Promicromonospora*, *Flavobacterium*, *Flavitalea*, *Ruminococcaceae NK4A214 group*, *Escherichia-Shigella*, *Anaerococcus*, *Gardnerella*, *Pediococcus*, *Roseiarcus*, *Kribbella*, *Methylosinus*, *Acetitomaculum*, *Brachymona*, *Iamia*, *Aerococcus*, *Nakamurella*, *Reyranella*, *Weissella*, *Moryella*, and *Shinella*. On the contrary, about 20 genera were detected only in tonsillar healthy status with sum relative abundance of 0.01%–0.03%. In addition, more active intra-microbiota correlations were found within all the three niches in tonsillar hypertrophy patients (**Figure 3**), reflected in both more positive and negative interactions among bacteria and more intense bacterial complex formation.

### Relationship Among Microbiota of Supragingival Plaque, Saliva, and Tonsil Surface Within Each Participant

Comparison of microbial diversities and compositions was made among the saliva, supragingival plaque, and tonsil surface microbiota in both tonsillar hypertrophic and healthy states, in order to gain insight into the microbiota of which niche in the oral cavity was more similar with the tonsil surface microbiota at different healthy states.

**Figure 4:**
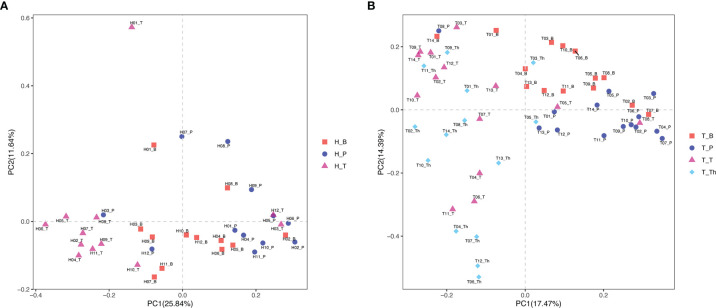
Principal coordinates analysis (PCoA) plot of microbiota community structure across different niches within tonsil healthy participants and tonsillar hypertrophy patients. **(A)** Microbiota community structure distribution in tonsil healthy participants. **(B)** Microbiota community structure distribution in tonsillar hypertrophy patients.

Results of alpha diversity analysis showed that the Shannon diversity of supragingival plaque microbiota was higher than that of tonsil surface microbiota in both healthy (*p* = 0.004) and tonsillar hypertrophic states (*p* = 0.037), while the Shannon diversity of salivary microbiota was higher than that of tonsil surface microbiota only in healthy condition (*p* = 0.011). No significant differences in other alpha diversity indexes were found in both conditions (**Figure 1**). PERMANOVA analysis of diversity based on the Bray–Curtis distance matrix showed significant differences among the microbiota of all the three niches (*p* < 0.01) in both healthy and tonsillar hypertrophic statuses (**Supplementary Table 4**). The F-model index of saliva and tonsil surface subgroups was smaller than that of supragingival plaque and tonsil surface subgroups in both healthy and tonsillar hypertrophic conditions, indicating that the microbiota construction of the tonsil surface was more similar with salivary microbiota, other than supragingival plaque microbiota. In addition, PCoA analysis showed that the dots representing the microbial composition of the salivary microbiota located closer to those of the tonsil surface microbiota in both states, compared with the supragingival plaque microbiota (**Figure 4**).

When coming to the microbial composition, the tonsil surface shared 76.9% and 81.3% of genera types with saliva in tonsillar healthy and hypertrophic statuses, respectively, with a total relative abundance of 99.92%–99.99%, while it shared 71.0% and 71.6% of genera types with supragingival plaque in the two states, respectively, with a total relative abundance of 99.86%–99.98% (**Figure 2**). These results indicated that the tonsil surface shared the most abundant content of microbiota with the oral cavity, no matter whether the tonsils are healthy or under hypertrophy conditions.

### Comparison of Microbiota of Tonsil Surface and Internal Tonsil Tissue Under the Condition of Tonsillar Hypertrophy

The diversity and composition of microbiota from the tonsil surface and internal tonsil tissue were compared in the tonsillar hypertrophy group in order to understand how representative the tonsil surface microbiota was of the overall tonsillar microbiota. The Chao1 and PD whole tree index were significantly higher in the tonsil surface than in the internal tonsil tissue microbiota (Chao1: *p* < 0.001, PD whole tree: *p* = 0.001). No significant differences were found in other alpha diversity indexes or any beta diversity indexes (*p* > 0.05) (**Figures 1**, **4**, and **Supplemental Table 4**).

About half of all the genera (104 in 241) that were detected from the tonsils were shared in both the tonsil surface and parenchyma, accounting for the majority of the tonsillar microbiota (99.60%–99.83%). However, the variety of genera on the tonsil surface was almost twice as many as that in the tonsil parenchyma. However, the particular genera in both sites only count for a minority of the total abundance (104 genera on the tonsil surface with total abundance of 0.40%; 33 in tonsil parenchyma with total abundance of 0.17%). Among the shared genera, *Rothia* and *Corynebacterium* exhibited significantly higher relative abundances in the microbiota of the tonsil surface than internal tonsil tissue (*p* < 0.01). (**Figure 2**) Less active within-microbiota interactions were detected in the internal tonsil tissue, with less active genera and less both positive and negative correlations. Moreover, the majority (83%) of the active genera were shared between both parenchyma and tonsil surface (**Figure 3**).

### Potential “Related Bacteria” in the Oral Microbiota for the Hypertrophic or Healthy Status of Tonsils

**Figure 5:**
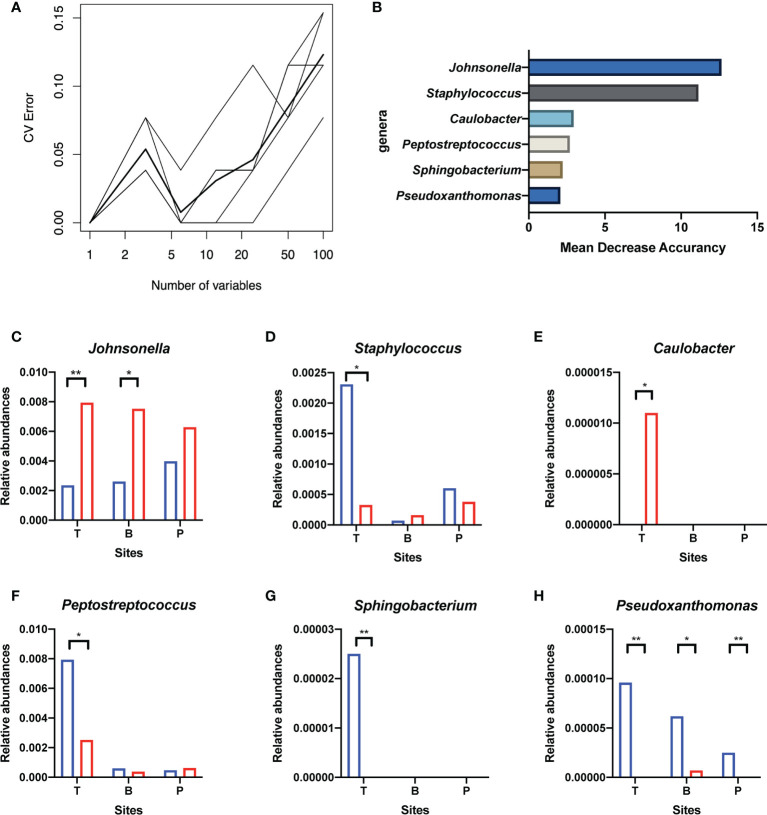
Screening of candidate “related bacteria” for the hypertrophic or healthy status of tonsils within the tonsil surface microbiota and their distribution in different niches. **(A)** The result of ten-fold cross-validations of the random forest model analysis within the tonsil surface microbiota. The ordinate is the prediction error rate, and the abscissa is the variable numbers; the thinner broken line is the result of each cross-check, and the thicker broken line is the average of the five repetitive cross-check results. In the figure, the abscissa value that meets criteria of greater than 1 and corresponds to the lowest error rate of the random forest model is six. That is, the number of bacteria that exert the greatest impact on the accuracy of the classified random forest model is six. **(B)** Top six genera of Mean Decrease Accuracy within the tonsil surface microbiota. Higher Mean Decrease Accuracy indicates higher the importance of the particular genus in the classification random forest model. The top 6 genera in the ranking of the Mean Decrease Accuracy value were selected as the candidate “related bacteria” for tonsillar hypertrophy and health status in the tonsil surface microbiota. **(C–H)** Distribution of the six candidate “related bacteria” in niches of tonsil surface, in saliva, and in supragingival plaque. *p < 0.05, **p < 0.01.

Genera of all three niches were ranked by importance index in random forest classification models. According to the result of ten-fold cross-validation, the top six genera in the microbiota of the tonsil surface were selected as candidate “related bacteria” for tonsil status, including *Johnsonella*, *Caulobacter*, *Staphylococcus*, *Peptostreptococcus*, *Sphingobacterium*, and *Pseudoxanthomonas* (**Figure 5**). Among them, *Johnsonella* and *Caulobacter* were significantly enriched in healthy condition (*p* < 0.05), while the other four genera exhibited the opposite trend of distribution (*p* < 0.05). In addition, *Pseudoxanthomonas* was significantly enriched on the tonsil surface than in the internal tonsil tissue (*p* = 0.034), while the relative abundances of other five genera exhibited no significant difference (*p* > 0.05).

The “related bacteria” in oral microbiota were then screened based on both relative abundance and importance index criteria. *Pseudoxanthomonas* was significantly enriched in the tonsillar hypertrophic condition in both salivary (*p* < 0.05) and supragingival plaque microbiota (*p* < 0.01), with importance indexes ranking within the top 22% and top 10% within the two niches, respectively. *Johnsonella* was significantly enriched in healthy condition in salivary microbiota (*p* < 0.05), with an importance index ranking within the top 1% (**Figure 5**). Based on these, we propose *Pseudoxanthomonas* as a potential “related bacteria” in both salivary and supragingival plaque microbiota for the condition of tonsillar hypertrophy and *Johnsonella* as a potential “related bacteria” in salivary microbiota for healthy tonsils.

## Discussion

In this study, we investigated the composition and relationship of oral and tonsillar microbiota in both healthy and tonsillar hypertrophic conditions in 3-to-6-year-old Chinese children. Variations of microbiota in saliva, in supragingival plaque, and on the tonsil surface were detected when tonsillar hypertrophy occurred. The microbial comparison of these three niches was also made to explore the similarity between oral and tonsillar microbiota within each participant. In addition, two genera in the oral cavity were screened out as potential “related bacteria” for healthy and hypertrophic status of the tonsils.

### Selection of Participants and Quality Control

Tonsils are the biggest peripheral immune organs in the oropharynx. The local immune function of tonsils becomes important after 3 years of age and will last until 10 years of age (Richardson, 1999). Tonsillar hypertrophy is a common disease among children. However, according to previous research, the composition and structure of oral microbiota vary a lot across ages and dentition stages (Crielaard et al., 2011). Around 6 years of age, the oral microbiota shifts markedly when the first permanent molars erupt, as the dentition stage changes from primary dentition to mixed dentition (Shi et al., 2016). In this study, we defined 3-to-6-year-old children with complete primary dentition as the inclusion criteria in order to minimize the impact of dentition variation on oral microbiota.

On the other hand, in accordance with the otolaryngological clinical guidelines, tonsillar hypertrophy is mainly treated by surgery of tonsillectomy, while a simple application of antibiotics is not recommended (Mitchell et al., 2019). However, misuse of antibiotics in children by parents or physicians cannot be completely avoided because the symptoms of tonsillitis sometimes are similar with upper respiratory tract infections (Bi et al., 2000; Chang et al., 2018). Antibiotics abuse can cause an increased risk of infection with drug-resistant bacteria and alteration in systemic microbiological structure (Chang et al., 2018). Considering these factors, we recruited children with no previous history of acute episodes of tonsillitis and no history of antibiotics use within the previous 1 month as the participants, in order to minimize the potential impact of previous antibiotics use on both oral and tonsillar microbiota.

In addition, results of questionnaire survey showed that tonsillar healthy children and tonsillar hypertrophy patients were well matched in terms of age, gender, maternal gestation and delivery, general health, family background, and oral hygiene habits. Oral examination revealed that caries morbidity and severity in both groups were of no significant difference and were similar to the average value at the particular age according to the results of 4th National Oral Health Survey in mainland China in 2018 (Wu Xiaoyan et al., 2019). Therefore, we consider that this study matched out well with the possible covariate impact factors, so as to focus on the characters of microbiota between oral cavity and tonsils in different health states.

### Comparison of the Tonsil Surface Microbiota With the Internal Tonsil Tissue Microbiota

In anatomy, the tonsils composed of lymphoid tissue that tightly bound to the epithelium and the internal epithelium of the tonsil descend into the tonsil parenchyma, forming cryptic foci within the tonsils. Swidsinski et al. (Swidsinski et al., 2007) found that bacteria within the tonsil parenchyma were mainly attached to the epithelial surface of the crypt and barely invaded the basal layer. For the convenience of sample collecting, microbial samples taken from the tonsil surface were widely used in previous studies of tonsillar microbiota (Khadilkar and Ankle, 2016; Wang et al., 2017; Galli et al., 2020; Katkowska et al., 2020).

In this study, in order to protect participants’ health interests and for ethical reasons, internal tissue of tonsils in healthy state could not be obtained. Therefore, we compared the surface and internal microbiota of tonsils in patients with tonsillar hypertrophy who needed tonsillectomy. Genera that existed in both surface and parenchyma of tonsils accounted for the majority of the tonsillar microbiota. The particular genera that were detected only in the parenchyma did not contribute much to the within-microbiota interactions. Based on these, we propose that the tonsil surface microbiota might be considered as a target sample source to represent the overall tonsillar microbiota when it comes to research that focus on the major microbial composition of tonsils.

### Relationship Between Oral Microbiota and Tonsil Surface Microbiota

The variation of oral and tonsil surface microbiota in tonsil hypertrophic state and the relationship among microbiota in different niches were the main focus of this study. The microbial diversity and dominant genera in the three niches all stayed stable when tonsillar hypertrophy occurred. More genera were detected in tonsillar hypertrophic status, but the additional genera constituted very low relative abundances. Among them, eleven genera were newly detected across all the three niches, most of which had been reported to be associated with infection or inflammation process. For instance, *Ruminococcus* and *Escherichia-Shigella* were detected in the inflammatory gut (Hall et al., 2017; Zhang et al., 2020), *Peptoniphilus* was detected in postoperative infected lesions in the oral cavity (Cho et al., 2015), *Gardnerella* was found to be associated with biofilm formation and bacterial vaginitis (Machado and Cerca, 2015), *Flavobacterium* and *Weissella* were thought to be opportunistic pathogens for bacteremia (Kamboj et al., 2015; Park and Ryoo, 2016), and *Bryobacter* was found to be associated with the accumulation of antibiotic resistance genes *in vitro* (Wan et al., 2021).

In addition, some other specific genera in the tonsillar hypertrophy participants had been reported to possess certain degree of antibiotics resistance, including *Herbaspirillum* (Bloise et al., 2020), *Chryseobacterium* (Im et al., 2020), *Sphingobacterium* (Barahona and Slim, 2015), and *Pseudoxanthomonas* (Chen Z. et al., 2018), which were detected on the tonsil surface, and *Stenotrophomonas* (Zhang et al., 2000), *Pseudohongiella* (Lv et al., 2020), and *Bryobacter* (Wan et al., 2021), which were detected in the oral microbiota. These results further indicated the potential role of antibiotic-resistant bacteria in the occurrence of tonsillar hypertrophy. It is possible that these specific bacteria may participate in the inflammation process and immune response in tonsillar hypertrophy. Moreover, more importantly, these results supported that a possible correlation might exist between shift in oral microbiota and the etiology of tonsillar hypertrophy. Within each individual, the microbiota structure within each niche was relatively independent, among which the microbiota of saliva and tonsil surface seemed more similar. This result was in accordance with research of Segata et al. (Segata et al., 2012) and may be related with the swallow path. During swallowing, saliva passes through the tonsils, and the bacteria within saliva may migrate onto the tonsil surface, leading to the similarity of the salivary microbiota and tonsil surface microbiota.

The main purpose of the co-occurrence analysis was to investigate the characters of bacterial interactions within each niche under both healthy and tonsil hypertrophy statuses. Previous studies have documented that when oral diseases such as caries and periodontal disease occur, the interactions within oral microbiota would increase markedly, with formation of bacterial complexes which exhibited strong correlations with each other, such as the “red” and “orange” complex that is found in patients of periodontal diseases (Socransky et al., 1998; Xu et al., 2018). Moreover, these microbial complexes were proposed to be considered as a driver for variations in community composition as well as indicators for the presence of diseases (Jakubovics, 2015; Gomez and Nelson, 2017). In this study, the bacterial correlations in the three niches all exhibited similar variation patterns from health to tonsil hypertrophy status, with more interactions and microbial complex formation. At the same time, the bacteria in complexes of each subgroup were all different, which also verified the independence of microbiota in each microenvironment. However, whether the bacterial genera in these complexes are representative or predictive for the disease state of the host still needs verification in further researches.

Multiple potential pathogenic bacteria were used to be detected in hyperplastic adenoids and palatine tonsils, but no causal link between the presence of these bacteria and hyperplasia has been established (Johnston and Douglas, 2018) Researchers found that certain species, such as *Streptococcus pyogenes*, *Haemophilus influenzae*, and *Moraxella catarrhalis*, generally colonized in the tonsillar crypts, and their interactions with other species played a determining role in their pathogenic actions (Galli et al., 2020). In our study, in order to further explore the relationship of oral microbiota with tonsil status, the random forest method (Breiman, 2001) was used, and according to the screening results, *Johnsonella* might be related with the healthy status of tonsils, and *Pseudoxanthomonas* might be related with the hypertrophic status of tonsils.

*Johnsonella* is a genus of anaerobic gram-negative bacteria that were originally detected in the human gingival sulcus in 1994, named after the American microbiologist John L. Johnson (Moore and Moore, 1994; Willems and Collins, 1995). *Johnsonella* can colonize in the oral cavity and on the tonsil surface as early as childhood and is a resident genus in both oral microbiota and tonsil surface microbiota (Jensen et al., 2013). Chen et al. found that *Johnsonella* was one of the resident genera in saliva and supragingival plaque in children aged 6–8 years (Chen et al., 2020). Jensen et al. found that *Johnsonella* was among the core genera of the tonsil surface microbiota in both healthy children aged 2–4 years and in children with recurrent tonsillitis (Jensen et al., 2013). Studies also showed that *Johnsonella* might be associated with periodontal disease in adults and oral squamous epithelial carcinoma (Huang et al., 2011; Chen C. et al., 2018) (Pushalkar et al., 2012). Up to now, no correlation between *Johnsonella* and tonsillar disease has been recorded, and its role in the inflammatory and immune response of the tonsils is still unclear.

*Pseudoxanthomonas* is often detected in the natural environment such as water, soil, compost, and plants (Kim et al., 2015; Lee et al., 2017; Mohapatra et al., 2018; Lin et al., 2019). It has also been reported in the bladder and intestine of children (Kispal et al., 2019). One study reported that *Pseudoxanthomonas* sp. *DIN-3* was effective in degrading non-steroidal anti-inflammatory drugs, including diclofenac, ibuprofen, and naproxen, in bioactive carbon filters (Lu et al., 2019). One *in vitro* mixed culture study found that *Pseudoxanthomonas* was associated with the degradation of ciprofloxacin (Liao et al., 2016). *Pseudoxanthomonas* may have the ability to spread antibiotic resistance genes (ARGs) in bacterial communities *in vitro* (Chen Z. et al., 2018). In 2018, a case report reported a chronic pericarditis in an adult patient caused by *Pseudoxanthomonas kaohsiungensis*, suggesting that this genus may have pathogenic ability under certain circumstances (Kuo and Lee, 2018).

In all, our study preliminarily explored the relationship between oral microbiome and tonsillar health states and screened out two candidate bacteria that may be closely related with the state of tonsils. Our findings may provide new directions for further research in the specific role of oral microbiota in the etiology of tonsillar hypertrophy and the tonsil immune response.

## Data Availability Statement

The data presented in the study are deposited in the NCBI repository, accession number PRJNA740113.

## Ethics Statement

The Ethics Committee of Peking University Health Science Center approved the study design, protocol, and informed consent procedure (PKUSSIRB-201840169). Written informed consents were received from parents or guardians of all the participants prior to enrollment.

## Author Contributions

Study design: MQ, HX, and BT. Sample collection: BT, XZ, and JZ. DNA extraction: BT. Analysis of the sequence data: BT, HX, WS, and JT. Paper preparation: BT and HX. Work conceiving and critically revising the manuscript: MQ. All authors contributed to the article and approved the submitted version.

## Funding

This work was supported by the Bureau of Health Care for Senior Officials of National Health and Family Planning Commission of China under Grant 220935; and Chinese National Program for Multidisciplinary Cooperative Treatment on Major Diseases under Grant PKUSSNMP-2020N.

## Conflict of Interest

The authors declare that the research was conducted in the absence of any commercial or financial relationships that could be construed as a potential conflict of interest.

## Publisher’s Note

All claims expressed in this article are solely those of the authors and do not necessarily represent those of their affiliated organizations, or those of the publisher, the editors and the reviewers. Any product that may be evaluated in this article, or claim that may be made by its manufacturer, is not guaranteed or endorsed by the publisher.

## References

[B1] Barahona F. Slim J. (2015). Sphingobacterium Multivorum: Case Report and Literature Review. New Microbes New Infect. 7, 33–36. doi: 10.1016/j.nmni.2015.04.006 26236492 PMC4501434

[B2] Bi P. Tong S. Parton K. A. (2000). Family Self-Medication and Antibiotics Abuse for Children and Juveniles in a Chinese City. Soc. Sci. Med. 50 (10), 1445–1450. doi: 10.1016/S0277-9536(99)00304-4 10741579

[B3] Bloise I. Guedez-López G. V. Tejedor-Rodríguez M. Romero-Gómez M. P. García-Rodríguez J. Mingorance J.. (2020). Bloodstream Infection Due to Herbaspirillum Sp.: Case Series and Review of Literature. Eur. J. Clin. Microbiol. Infect. Dis 40(4), 779–85. doi: 10.1007/s10096-020-04075-4 33083918

[B4] Bolger A. M. Lohse M. Usadel B. (2014). Trimmomatic: A Flexible Trimmer for Illumina Sequence Data. Bioinformatics 30 (15), 2114–2120. doi: 10.1093/bioinformatics/btu170 24695404 PMC4103590

[B5] Breiman L. (2001). Random Forest. Mach. Learn. 45, 5–32. doi: 10.1023/A:1010933404324

[B6] Caporaso J. G. Kuczynski J. Stombaugh J. Bittinger K. Bushman F. D. Costello E. K.. (2010). QIIME Allows Analysis of High-Throughput Community Sequencing Data. Nat. Methods 7 (5), 335–336. doi: 10.1038/nmeth.f.303 20383131 PMC3156573

[B7] Casarin R. C. Barbagallo A. Meulman T. Santos V. R. Sallum E. A. Nociti F. H.. (2013). Subgingival Biodiversity in Subjects With Uncontrolled Type-2 Diabetes and Chronic Periodontitis. J. Periodontal. Res. 48 (1), 30–36. doi: 10.1111/j.1600-0765.2012.01498.x 22762355

[B8] Chang J. Lv B. Zhu S. Yu J. Zhang Y. Ye D.. (2018). Non-Prescription Use of Antibiotics Among Children in Urban China: A Cross-Sectional Survey of Knowledge, Attitudes, and Practices. Expert Rev. Anti Infect. Ther. 16 (2), 163–172. doi: 10.1080/14787210.2018.1425616 29310469

[B9] Cheng M. Zhang X. Zhu J. Cheng L. Cao J. Wu Z.. (2018). A Metagenomics Approach to the Intestinal Microbiome Structure and Function in High Fat Diet-Induced Obesity Mice Fed With Oolong Tea Polyphenols. Food Funct. 9 (2), 1079–1087. doi: 10.1039/c7fo01570d 29355278

[B10] Chen C. Hemme C. Beleno J. Shi Z. J. Ning D. Qin Y.. (2018). Oral Microbiota of Periodontal Health and Disease and Their Changes After Nonsurgical Periodontal Therapy. Isme. J. 12 (5), 1210–1224. doi: 10.1038/s41396-017-0037-1 29339824 PMC5932080

[B11] Chen W. Jiang Q. Yan G. Yang D. (2020). The Oral Microbiome and Salivary Proteins Influence Caries in Children Aged 6 to 8 Years. BMC Oral. Health 20 (1), 295. doi: 10.1186/s12903-020-01262-9 33115458 PMC7592381

[B12] Chen Z. Wang Y. Wen Q. (2018). Effects of Chlortetracycline on the Fate of Multi-Antibiotic Resistance Genes and the Microbial Community During Swine Manure Composting. Environ. Pollut. 237, 977–987. doi: 10.1016/j.envpol.2017.11.009 29137887

[B13] Chen M. Wu B. L. Chen T. Liu Z. Deng Z. L. Peng L. (2016). The Impact of Different DNA Extraction Methods on the Analysis of Microbial Diversity of Oral Saliva From Healthy Youths by Polymerase Chain Reaction-Denaturing Gradient Gel Electrophoresis. J. Dent. Sci. 11 (1), 54–58. doi: 10.1016/j.jds.2015.08.002 30894946 PMC6395153

[B14] Cho E. Park S. N. Shin Y. Lim Y. K. Paek J. Kim H. K.. (2015). Peptoniphilus Mikwangii Sp. Nov., Isolated From a Clinical Specimen of Human Origin. Curr. Microbiol. 70 (2), 260–266. doi: 10.1007/s00284-014-0712-7 25319028

[B15] Crielaard W. Zaura E. Schuller A. A. Huse S. M. Montijn R. C. Keijser B. J. (2011). Exploring the Oral Microbiota of Children at Various Developmental Stages of Their Dentition in the Relation to Their Oral Health. BMC Med. Genomics 4:22. doi: 10.1186/1755-8794-4-22 21371338 PMC3058002

[B16] de Smit M. J. Brouwer E. Vissink A. van Winkelhoff A. J. (2011). Rheumatoid Arthritis and Periodontitis; a Possible Link via Citrullination. Anaerobe 17 (4), 196–200. doi: 10.1016/j.anaerobe.2011.03.019 21515392

[B17] Edgar R. C. (2013). UPARSE: Highly Accurate OTU Sequences From Microbial Amplicon Reads. Nat. Methods 10 (10), 996–998. doi: 10.1038/nmeth.2604 23955772

[B18] Edgar R. C. Haas B. J. Clemente J. C. Quince C. Knight R. (2011). UCHIME Improves Sensitivity and Speed of Chimera Detection. Bioinformatics 27 (16), 2194–2200. doi: 10.1093/bioinformatics/btr381 21700674 PMC3150044

[B19] Galli J. Calò L. Posteraro B. Rossi G. Sterbini F. P. Paludetti G.. (2020). Pediatric Oropharyngeal Microbiome: Mapping in Chronic Tonsillitis and Tonsillar Hypertrophy. Int. J. Pediatr. Otorhinolaryngol. 139, 110478. doi: 10.1016/j.ijporl.2020.110478 33160244

[B20] Gao Shuwei G. Y. Xu Z. (2020). Research Progress on the Predictive Factors for the Outcomes of Adenotonsillectomy in Children With Obstructive Sleep Apnea Syndrome. Chin. J. Otorhinolaryngol. Head Neck Surg. 55 (06), 630–634. doi: 10.3760/cma.j.cn115330-20191105-00666 32610410

[B21] Gomez A. Nelson K. E. (2017). The Oral Microbiome of Children: Development, Disease, and Implications Beyond Oral Health. Microb. Ecol. 73 (2), 492–503. doi: 10.1007/s00248-016-0854-1 27628595 PMC5274568

[B22] Hall A. B. Yassour M. Sauk J. Garner A. Jiang X. Arthur T.. (2017). A Novel Ruminococcus Gnavus Clade Enriched in Inflammatory Bowel Disease Patients. Genome Med. 9 (1), 103. doi: 10.1186/s13073-017-0490-5 29183332 PMC5704459

[B23] Han Y. W. Wang X. (2013). Mobile Microbiome: Oral Bacteria in Extra-Oral Infections and Inflammation. J. Dental Res. 92 (6), 485–491. doi: 10.1177/0022034513487559 PMC3654760 23625375

[B24] Huang S. Yang F. Zeng X. Chen J. Li R. Wen T.. (2011). Preliminary Characterization of the Oral Microbiota of Chinese Adults With and Without Gingivitis. BMC Oral. Health 11 (1), 33. doi: 10.1186/1472-6831-11-33 22152152 PMC3254127

[B25] Im J. H. Kim D. Kim J. J. Kim E. Y. Park Y. K. Kwon H. Y.. (2020). Chryseobacterium Arthrosphaerae Ventriculitis: A Case Report. Med. (Baltimore). 99 (34), e21751. doi: 10.1097/md.0000000000021751 PMC7447447 32846799

[B26] Jakubovics N. S. (2015). Intermicrobial Interactions as a Driver for Community Composition and Stratification of Oral Biofilms. J. Mol. Biol. 427 (23), 3662–3675. doi: 10.1016/j.jmb.2015.09.022 26519790

[B27] Jensen A. Fagö-Olsen H. Sørensen C. H. Kilian M. (2013). Molecular Mapping to Species Level of the Tonsillar Crypt Microbiota Associated With Health and Recurrent Tonsillitis. PLoS One 8 (2), e56418. doi: 10.1371/journal.pone.0056418 23437130 PMC3578847

[B28] Johnston J. J. Douglas R. (2018). Adenotonsillar Microbiome: An Update. Postgraduate. Med. J. 94 (1113), 398–403. doi: 10.1136/postgradmedj-2018-135602 29884749

[B29] Kamboj K. Vasquez A. Balada-Llasat J. M. (2015). Identification and Significance of Weissella Species Infections. Front. Microbiol. 6, 1204. doi: 10.3389/fmicb.2015.01204 26583007 PMC4628101

[B30] Katkowska M. Garbacz K. Kopala W. Schubert J. Bania J. (2020). Genetic Diversity and Antimicrobial Resistance of Staphylococcus Aureus From Recurrent Tonsillitis in Children. Apmis 128 (3), 211–219. doi: 10.1111/apm.13007 31692060

[B31] Khadilkar M. N. Ankle N. R. (2016). Anaerobic Bacteriological Microbiota in Surface and Core of Tonsils in Chronic Tonsillitis. J. Clin. Diagn. Res. 10 (11), Mc01–mc03. doi: 10.7860/jcdr/2016/22124.8819 PMC5198365 28050412

[B32] Kim S. J. Ahn J. H. Weon H. Y. Lim J. M. Kim S. G. Kwon S. W. (2015). Pseudoxanthomonas Sangjuensis Sp. Nov., Isolated From Greenhouse Soil. Int. J. Syst. Evol. Microbiol. 65 (9), 3170–3174. doi: 10.1099/ijsem.0.000395 26297383

[B33] Kispal Z. F. Vajda P. Kardos D. Klymiuk I. Moissl-Eichinger C. Castellani C.. (2019). The Local Microbiome After Pediatric Bladder Augmentation: Intestinal Segments and the Native Urinary Bladder Host Similar Mucosal Microbiota. J. Pediatr. Urol. 15 (1), 30.e31–30.e37. doi: 10.1016/j.jpurol.2018.07.028 30206025

[B34] Kuo S. F. Lee C. H. (2018). An Oil Refinery Worker at Kaohsiung, With Pseudoxanthomonas Kaohsiungensis Bloodstream Infection Presenting as Chronic Pericarditis and Masquerading as Tuberculosis Pericarditis. J. Microbiol. Immunol. Infect. 51 (4), 575–577. doi: 10.1016/j.jmii.2017.12.003 29366692

[B35] Kuwabara M. Motoki Y. Ichiura K. Fujii M. Inomata C. Sato H.. (2016). Association Between Toothbrushing and Risk Factors for Cardiovascular Disease: A Large-Scale, Cross-Sectional Japanese Study. BMJ Open 6 (1), e009870. doi: 10.1136/bmjopen-2015-009870 PMC4735199 26769787

[B36] Lee J. K. Oh J. S. Cho W. D. Roh D. H. (2017). Pseudoxanthomonas Putridarboris Sp. Nov. Isolated From Rotten Tree. Int. J. Syst. Evol. Microbiol. 67 (6), 1807–1812. doi: 10.1099/ijsem.0.001867 28598308

[B37] Liao X. Li B. Zou R. Dai Y. Xie S. Yuan B. (2016). Biodegradation of Antibiotic Ciprofloxacin: Pathways, Influential Factors, and Bacterial Community Structure. Environ. Sci. Pollut. Res. Int. 23 (8), 7911–7918. doi: 10.1007/s11356-016-6054-1 26762935

[B38] Li X. Kolltveit K. M. Tronstad L. Olsen I. (2000). Systemic Diseases Caused by Oral Infection. Clin. Microbiol. Rev. 13 (4), 547–558. doi: 10.1128/cmr.13.4.547 11023956 PMC88948

[B39] Lin J. Yang G. Tang J. Li Z. Yu Z. Zhuang L. (2019). Pseudoxanthomonas Composti Sp. Nov., Isolated From Compost. Antonie. Van. Leeuwenhoek. 112 (8), 1213–1219. doi: 10.1007/s10482-019-01253-z 30852702

[B40] Lu Z. Sun W. Li C. Ao X. Yang C. Li S. (2019). Bioremoval of non-Steroidal Anti-Inflammatory Drugs by Pseudoxanthomonas Sp. DIN-3 Isolated From Biological Activated Carbon Process. Water Res. 161, 459–472. doi: 10.1016/j.watres.2019.05.065 31229727

[B41] Lv B. Cui Y. Tian W. Wei H. Chen Q. Liu B.. (2020). Vessel Transport of Antibiotic Resistance Genes Across Oceans and its Implications for Ballast Water Management. Chemosphere 253, 126697. doi: 10.1016/j.chemosphere.2020.126697 32298915

[B42] Machado A. Cerca N. (2015). Influence of Biofilm Formation by Gardnerella Vaginalis and Other Anaerobes on Bacterial Vaginosis. J. Infect. Dis. 212 (12), 1856–1861. doi: 10.1093/infdis/jiv338 26080369

[B43] Mitchell R. B. Archer S. M. Ishman S. L. Rosenfeld R. M. Coles S. Finestone S. A.. (2019). Clinical Practice Guideline: Tonsillectomy in Children (Update). Otolaryngol. Head Neck Surg. 160 (1_suppl), S1–s42. doi: 10.1177/0194599818801757 30798778

[B44] Mohapatra B. Sar P. Kazy S. K. Maiti M. K. Satyanarayana T. (2018). Taxonomy and Physiology of Pseudoxanthomonas Arseniciresistens Sp. Nov., an Arsenate and Nitrate-Reducing Novel Gammaproteobacterium From Arsenic Contaminated Groundwater, India. PLoS One 13 (3), e0193718. doi: 10.1371/journal.pone.0193718 29558470 PMC5860741

[B45] Moore L. V. Moore W. E. (1994). Oribaculum Catoniae Gen. Nov., Sp. Nov.; Catonella Morbi Gen. Nov., Sp. Nov.; Hallella Seregens Gen. Nov., Sp. Nov.; Johnsonella Ignava Gen. Nov., Sp. Nov.; and Dialister Pneumosintes Gen. Nov., Comb. Nov., Nom. Rev., Anaerobic Gram-Negative Bacilli From the Human Gingival Crevice. Int. J. Syst. Bacteriol. 44 (2), 187–192. doi: 10.1099/00207713-44-2-187 8186083

[B46] Mora R. Salami A. Passali F. M. Mora F. Cordone M. P. Ottoboni S.. (2003). OSAS in Children. Int. J. Pediatr. Otorhinolaryngol. 67 (Suppl 1), S229–S231. doi: 10.1016/j.ijporl.2003.08.034 14662202

[B47] Park S. K. Ryoo N. (2016). A Case of Flavobacterium Ceti Meningitis. Ann. Lab. Med. 36 (6), 614–616. doi: 10.3343/alm.2016.36.6.614 27578519 PMC5011119

[B48] Pushalkar S. Ji X. Li Y. Estilo C. Yegnanarayana R. Singh B.. (2012). Comparison of Oral Microbiota in Tumor and non-Tumor Tissues of Patients With Oral Squamous Cell Carcinoma. BMC Microbiol. 12, 144. doi: 10.1186/1471-2180-12-144 22817758 PMC3507910

[B49] Rajasuo A. Leppanen J. Savolainen S. Meurman J. H. (1996). Pericoronitis and Tonsillitis: Clinical and Darkfield Microscopy Findings. Oral. Surg. Oral. Med. Oral. Pathol. Oral. Radiol. Endod. 81 (5), 526–532. doi: 10.1016/S1079-2104(96)80041-2 8734697

[B50] Richardson M. A. (1999). Sore Throat, Tonsillitis, and Adenoiditis. Med. Clin. North Am. 83 75-83 (1), viii. doi: 10.1016/s0025-7125(05)70088-2 9927961

[B51] Riviere G. R. Riviere K. H. Smith K. S. (2002). Molecular and Immunological Evidence of Oral Treponema in the Human Brain and Their Association With Alzheimer's Disease. Oral. Microbiol. Immunol. 17 (2), 113–118. doi: 10.1046/j.0902-0055.2001.00100.x 11929559

[B52] Segata N. Haake S. K. Mannon P. Lemon K. P. Waldron L. Gevers D.. (2012). Composition of the Adult Digestive Tract Bacterial Microbiome Based on Seven Mouth Surfaces, Tonsils, Throat and Stool Samples. Genome Biol. 13 (6), R42. doi: 10.1186/gb-2012-13-6-r42 22698087 PMC3446314

[B53] Shi W. Qin M. Chen F. Xia B. (2016). Supragingival Microbial Profiles of Permanent and Deciduous Teeth in Children With Mixed Dentition. PLoS One 11 (1), e0146938. doi: 10.1371/journal.pone.0146938 26752284 PMC4709228

[B54] Socransky S. S. Haffajee A. D. Cugini M. A. Smith C. Kent R. L. Jr. (1998). Microbial Complexes in Subgingival Plaque. J. Clin. Periodontol. 25 (2), 134–144. doi: 10.1111/j.1600-051x.1998.tb02419.x 9495612

[B55] Souza J. F. Grechi T. H. Anselmo-Lima W. T. Trawitzki L. V. Valera F. C. (2013). Mastication and Deglutition Changes in Children With Tonsillar Hypertrophy. Braz. J. Otorhinolaryngol. 79 (4), 424–428. doi: 10.5935/1808-8694.20130076 23929140 PMC9442428

[B56] Soylu E. Soylu N. Polat C. Sakallıoğlu Ö. Uçur Ö. Bozdoğan G. (2016). Developmental Delays in Preschool Children With Adenotonsillar Hypertrophy. Kulak. Burun. Bogaz. Ihtis. Derg. 26 (3), 129–134. doi: 10.5606/kbbihtisas.2016.42724 27107598

[B57] Spahr A. Klein E. Khuseyinova N. Boeckh C. Muche R. Kunze M.. (2006). Periodontal Infections and Coronary Heart Disease. Arch. Intern. Med. 166 (5), 554. doi: 10.1001/archinte.166.5.554 16534043

[B58] Swidsinski A. Göktas Ö. Bessler C. Loening-Baucke V. Hale L. P. Andree H.. (2007). Spatial Organisation of Microbiota in Quiescent Adenoiditis and Tonsillitis. J. Clin. Pathol. 60 (3), 253. doi: 10.1136/jcp.2006.037309 16698947 PMC1860565

[B59] Wang H. Dai W. Feng X. Zhou Q. Wang H. Yang Y.. (2018). Microbiota Composition in Upper Respiratory Tracts of Healthy Children in Shenzhen, China, Differed With Respiratory Sites and Ages. BioMed. Res. Int. 2018, 6515670. doi: 10.1155/2018/6515670 30013985 PMC6022278

[B60] Wang Q. Du J. Jie C. Ouyang H. Luo R. Li W. (2017). Bacteriology and Antibiotic Sensitivity of Tonsillar Diseases in Chinese Children. Eur. Arch. Oto-Rhino-Laryngol. 274 (8), 3153–3159. doi: 10.1007/s00405-017-4603-y 28551703

[B61] Wan K. Guo L. Ye C. Zhu J. Zhang M. Yu X. (2021). Accumulation of Antibiotic Resistance Genes in Full-Scale Drinking Water Biological Activated Carbon (BAC) Filters During Backwash Cycles. Water Res. 190, 116744. doi: 10.1016/j.watres.2020.116744 33401101

[B62] WHO (1997). Oral Health Surveys: Basic Methods. 3rd Edn (Geneva: World Health Organization).

[B63] Willems A. Collins M. D. (1995). Evidence for the Placement of the Gram-Negative Catonella Morbi (Moore and Moore) and Johnsonella Ignava (Moore and Moore) Within the Clostridium Subphylum of the Gram-Positive Bacteria on the Basis of 16S rRNA Sequences. Int. J. Syst. Bacteriol. 45 (4), 855–857. doi: 10.1099/00207713-45-4-855 7547310

[B64] Wu X. Y. Wang J. X. Cai T. Li Y. Zhou Z. Yang Z. (2019). Prevalence and Influencing Factors of Deciduous Caries in Preschool Children in Chongqing City. West. China J. Stomatol. 37 (1), 81–85. doi: 10.7518/hxkq.2019.01.016 PMC7030743 30854825

[B65] Xue X. C. Chen X. P. Yao W. H. Zhang Y. Sun G. B. Tan X. J. (2014). Prevalence of Human Papillomavirus and Epstein-Barr Virus DNA in Chinese Children With Tonsillar and/or Adenoidal Hypertrophy. J. Med. Virol. 86 (6), 963–967. doi: 10.1002/jmv.23894 24615954

[B66] Xun Z. Zhang Q. Xu T. Chen N. Chen F. (2018). Dysbiosis and Ecotypes of the Salivary Microbiome Associated With Inflammatory Bowel Diseases and the Assistance in Diagnosis of Diseases Using Oral Bacterial Profiles. Front. Microbiol. 9, 1136. doi: 10.3389/fmicb.2018.01136 29899737 PMC5988890

[B67] Xu H. Tian J. Hao W. Zhang Q. Zhou Q. Shi W.. (2018). Oral Microbiome Shifts From Caries-Free to Caries-Affected Status in 3-Year-Old Chinese Children: A Longitudinal Study. Front. Microbiol. 9, 2009. doi: 10.3389/fmicb.2018.02009 30210479 PMC6121080

[B68] Yeoh Y. K. Chan M. H. Chen Z. Lam E. W. H. Wong P. Y. Ngai C. M.. (2019). The Human Oral Cavity Microbiota Composition During Acute Tonsillitis: A Cross-Sectional Survey. BMC Oral. Health 19 (1), 275. doi: 10.1186/s12903-019-0956-5 31806002 PMC6896734

[B69] Zhang J. Kobert K. Flouri T. Stamatakis A. (2014). PEAR: A Fast and Accurate Illumina Paired-End reAd mergeR. Bioinformatics 30 (5), 614–620. doi: 10.1093/bioinformatics/btt593 24142950 PMC3933873

[B70] Zhang L. Li X. Z. Poole K. (2000). Multiple Antibiotic Resistance in Stenotrophomonas Maltophilia: Involvement of a Multidrug Efflux System. Antimicrobial. Agents Chemother. 44 (2), 287–293. doi: 10.1128/AAC.44.2.287-293.2000 PMC89673 10639352

[B71] Zhang Z. Taylor L. Shommu N. Ghosh S. Reimer R. Panaccione R.. (2020). A Diversified Dietary Pattern Is Associated With a Balanced Gut Microbial Composition of Faecalibacterium and Escherichia/Shigella in Patients With Crohn's Disease in Remission. J. Crohns. Colitis. 14 (11), 1547–1557. doi: 10.1093/ecco-jcc/jjaa084 32343765

[B72] Zhang Z. Zhai H. Geng J. Yu R. Ren H. Fan H.. (2013). Large-Scale Survey of Gut Microbiota Associated With MHE Via 16s rRNA-Based Pyrosequencing. Am. J. Gastroenterol. 108 (10), 1601–1611. doi: 10.1038/ajg.2013.221 23877352

