# Supplementary material for: A Correlation Study of the Microbiota Between Oral Cavity and Tonsils in Children With Tonsillar Hypertrophy
Source: Front Cell Infect Microbiol. 2022 Jan 28;11:724142. doi: 10.3389/fcimb.2021.724142 (PMC8831826; doi:10.3389/fcimb.2021.724142)
Supplement: Supplementary file 1 [file Image_1.pdf]

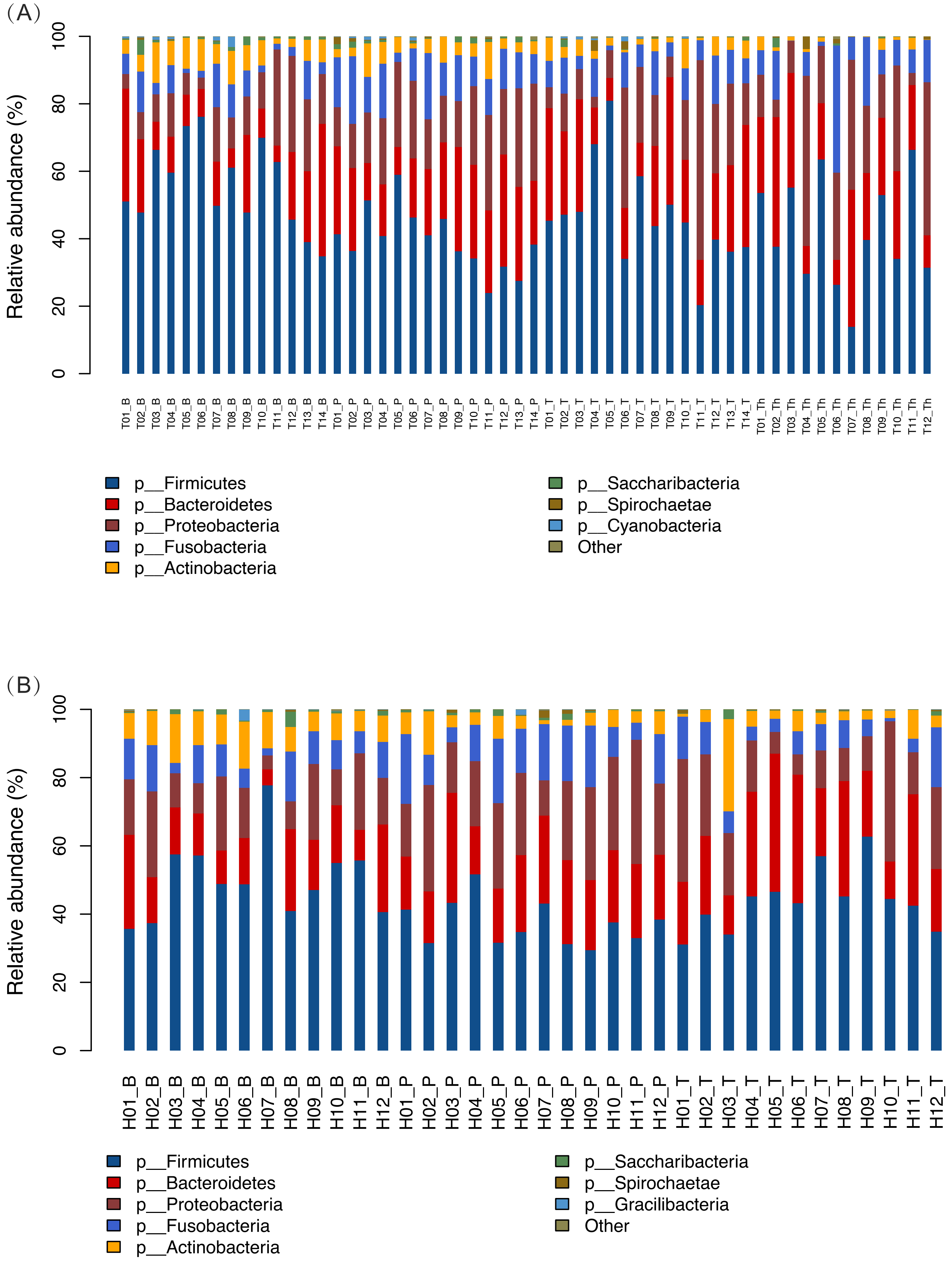

Supplementary Figure 1: Boxplots of Relative Abundance at Phylum Level. (A) tonsillar hypertrophy group, (B) control group
