# Supplementary material for: A Correlation Study of the Microbiota Between Oral Cavity and Tonsils in Children With Tonsillar Hypertrophy
Source: Front Cell Infect Microbiol. 2022 Jan 28;11:724142. doi: 10.3389/fcimb.2021.724142 (PMC8831826; doi:10.3389/fcimb.2021.724142)
Supplement: Supplementary file 2 [file Table_1.docx]

Supplementary Table 1

# Supplementary Table 1 Gender, age and caries status of study participants

| Groups | Number of Children | Gender | | Age  （Mean±SD） | dmfs  （Mean±SD） | dmft  （Mean±SD） |
| --- | --- | --- | --- | --- | --- | --- |
|  |  | Male | Female |  |  |  |
| T | 14 | 12 | 2 | 4.7±0.9 | 6.1±5.9 | 4.1±3.6 |
| H | 12 | 6 | 6 | 4.6±1 | 7.1±6.7 | 5.4±4.2 |
| χ^2^ |  | 3.720 | | 0.073 | 0.230 | 0.831 |
| *P* |  | 0.054 | | 0.787 | 0.632 | 0.362 |
