# Supplementary material for: A Correlation Study of the Microbiota Between Oral Cavity and Tonsils in Children With Tonsillar Hypertrophy
Source: Front Cell Infect Microbiol. 2022 Jan 28;11:724142. doi: 10.3389/fcimb.2021.724142 (PMC8831826; doi:10.3389/fcimb.2021.724142)
Supplement: Supplementary file 3 [file Table_2.docx]

Supplementary Table 2

# Supplementary Table 2 Results of the questionnaire

Supplementary Table 2(a) Maternal gestation and delivery status of the mothers

| Groups | Number of children | Health status of the mother during maternal gestation | | | | Mode of delivery | | Birth weight | | | Birth length | | |
| --- | --- | --- | --- | --- | --- | --- | --- | --- | --- | --- | --- | --- | --- |
|  |  | Pregnancy-induced hypertension | Gestational Diabetes | Both | Healthy | Spontaneous delivery | Cesarean section | Normal | High | Low | Normal | High | Low |
| T | 14 | 0 | 2 | 0 | 12 | 7 | 7 | 13 | 0 | 1 | 13 | 1 | 0 |
| H | 12 | 1 | 3 | 0 | 8 | 8 | 4 | 12 | 0 | 0 | 11 | 0 | 1 |
| χ^2^ |  | 1.420 | | | | 0.707 | | 1.905 | | | 0.647 | | |
| *P* |  | 0.233 | | | | 0.400 | | 0.167 | | | 0.421 | | |

Supplementary Table 2(b) General health status and family background

| Groups | Number of children | BMI | | | | History of major diseases | | Caregivers | | | Educational level of father | | |
| --- | --- | --- | --- | --- | --- | --- | --- | --- | --- | --- | --- | --- | --- |
|  |  | Normal | Overweight | Obesity | Underweight | Present | None | Parents | Parents and grandparents | Others | Junior high school or lower | High school | College or higher |
| T | 14 | 6 | 1 | 0 | 7 | 1 | 13 | 11 | 3 | 0 | 1 | 3 | 10 |
| H | 12 | 3 | 2 | 3 | 4 | 1 | 11 | 6 | 6 | 0 | 0 | 2 | 10 |
| χ^2^ |  | 2.525 | | | | 0.012 | | 2.241 | | | 0.594 | | |
| *P* |  | 0.112 | | | | 0.911 | | 0.134 | | | 0.441 | | |

Supplementary Table 2(c) Family background (cont.)

| Groups | Number of children | Educational level of mother | | | | Living environment | | | Regular physical examinations | |
| --- | --- | --- | --- | --- | --- | --- | --- | --- | --- | --- |
|  |  | Primary school | Junior high school | High school | College or higher | City | Township | Rural | Yes | No |
| T | 14 | 1 | 1 | 2 | 10 | 12 | 1 | 1 | 9 | 5 |
| H | 12 | 0 | 1 | 3 | 8 | 9 | 3 | 0 | 8 | 4 |
| χ^2^ |  | 0.009 | | | | 0.317 | | | 0.016 | |
| *P* |  | 0.924 | | | | 0.574 | | | 0.901 | |

Supplementary Table 2(d) Oral hygiene practices

| Groups | Number of children | Method of toothbrushing | | | | Brushing frequency | | | | Time of toothbrushing | | | |
| --- | --- | --- | --- | --- | --- | --- | --- | --- | --- | --- | --- | --- | --- |
|  |  | By parents | By the child | By the child and supervised by parents | Never | Twice a day | Once a day | Occasionally | Never | <1min | 1-2min | > 2min | Never |
| T | 14 | 5 | 4 | 5 | 0 | 7 | 7 | 0 | 0 | 4 | 8 | 2 | 0 |
| H | 12 | 3 | 6 | 2 | 1 | 6 | 3 | 0 | 1 | 1 | 8 | 2 | 1 |
| χ^2^ |  | 0.018 | | | | 0.375 | | | | 0.224 | | | |
| *P* |  | 0.892 | | | | 0.540 | | | | 0.636 | | | |

Supplementary Table 2(e) Oral hygiene practices (cont.)

| Groups | Number of children | Tools of toothbrushing | | | | Fluoridated toothpaste | | Frequency of using dental floss | | |
| --- | --- | --- | --- | --- | --- | --- | --- | --- | --- | --- |
|  |  | Manual toothbrushes | Electric toothbrush | Both | Never | Yes | No or not sure | At least once a day | Occasionally | Never |
| T | 14 | 11 | 2 | 1 | 0 | 2 | 12 | 0 | 2 | 12 |
| H | 12 | 8 | 2 | 1 | 1 | 6 | 6 | 1 | 1 | 10 |
| χ^2^ |  | 0.529 | | | | 3.720 | | 0.061 | | |
| *P* |  | 0.467 | | | | 0.054 | | 0.806 | | |
