# Supplementary material for: A Correlation Study of the Microbiota Between Oral Cavity and Tonsils in Children With Tonsillar Hypertrophy
Source: Front Cell Infect Microbiol. 2022 Jan 28;11:724142. doi: 10.3389/fcimb.2021.724142 (PMC8831826; doi:10.3389/fcimb.2021.724142)
Supplement: Supplementary file 4 [file Table_3.docx]

Supplementary Table 3

# Supplementary Table 3 Average Relative Abundance (%) of Dominant Genera in All Subgroups

| Genus | Relative Abundance (%) | | | | | | |
| --- | --- | --- | --- | --- | --- | --- | --- |
|  | H_B | H_P | H_T | T_B | T_P | T_T | T_Th |
| *Streptococcus* | 28.5 | 21.3 | 23.0 | 34.8 | 21.1 | 22.4 | 13.6 |
| *Neisseria* | 7.7 | 11.9 | 10.1 | 5.1 | 10.1 | 4.9 | 5.5 |
| *Prevotella_7* | 2.5 | 3.1 | 9.0 | 5.6 | 3.0 | 8.9 | 5.6 |
| *Veillonella* | 4.2 | 4.0 | 5.6 | 3.5 | 6.3 | 7.3 | 6.6 |
| *Gemella* | 6.7 | 2.8 | 5.3 | 8.2 | 2.9 | 5.6 | 4.7 |
| *Haemophilus* | 2.5 | 3.2 | 5.0 | 4.4 | 2.3 | 9.5 | 13.8 |
| *Porphyromonas* | 3.9 | 4.9 | 4.3 | 3.3 | 5.3 | 3.6 | 5.3 |
| *Prevotella* | 2.3 | 1.4 | 4.2 | 2.2 | 2.6 | 3.0 | 3.0 |
| *Fusobacterium* | 4.0 | 5.5 | 4.6 | 2.8 | 5.8 | 5.7 | 7.4 |
| *Leptotrichia* | 4.8 | 7.4 | 2.4 | 3.2 | 6.0 | 2.6 | 2.7 |
