# Supplementary material for: A Correlation Study of the Microbiota Between Oral Cavity and Tonsils in Children With Tonsillar Hypertrophy
Source: Front Cell Infect Microbiol. 2022 Jan 28;11:724142. doi: 10.3389/fcimb.2021.724142 (PMC8831826; doi:10.3389/fcimb.2021.724142)
Supplement: Supplementary file 5 [file Table_4.docx]

***Supplementary Table 4***

**Supplementary Table 4 PERMANOVA Analysis of Microbial Community Composition**

|  | Sums of squares | Mean squares | F. Model | Variation (R^2^) | *P* value ^a^ |
| --- | --- | --- | --- | --- | --- |
| H_B & T_B | 0.170 | 0.170 | 1.294 | 0.051 | 0.203 |
| H_P & T_P | 0.101 | 0.101 | 0.778 | 0.031 | 0.075 |
| H_T & T_T | 0.114 | 0.114 | 0.586 | 0.024 | 0.899 |
| H_B & H_P | 0.257 | 0.257 | 2.132 | 0.068 | 0.009** |
| H_B & H_T | 0.383 | 0.383 | 2.579 | 0.105 | 0.009** |
| H_P & H_T | 0.592 | 0.592 | 3.922 | 0.151 | 0.006** |
| T_B & T_P | 0.634 | 0.634 | 4.376 | 0.144 | 0.002** |
| T_B & T_T | 0.535 | 0.535 | 3.047 | 0.105 | 0.005** |
| T_B & T_Th | 0.990 | 0.990 | 4.901 | 0.159 | 0.002** |
| T_P & T_T | 0.950 | 0.950 | 5.496 | 0.175 | 0.002** |
| T_P & T_Th | 1.121 | 1.121 | 5.631 | 0.178 | 0.002** |
| T_T & T_Th | 0.294 | 0.294 | 1.277 | 0.047 | 0.181 |

^a^ *0.01<P<0.05, **0.001<p<0.01, ***P<0.001
