# Supplementary material for: A Correlation Study of the Microbiota Between Oral Cavity and Tonsils in Children With Tonsillar Hypertrophy
Source: Front Cell Infect Microbiol. 2022 Jan 28;11:724142. doi: 10.3389/fcimb.2021.724142 (PMC8831826; doi:10.3389/fcimb.2021.724142)
Supplement: Supplementary file 6 [file Table_5.docx]

Supplementary Table 5

# Supplementary Table 5 Genera Detected only in Tonsillar Hypertrophy Patients or only in Control Participants

| Site | Group | Genera |
| --- | --- | --- |
| Tonsillar surface | T | *Dyella, Massilia, Cnuella, Sphingopyxis, Pseudarthrobacter, Proteiniphilum, Arthrobacter, Nocardioides, Pseudonocardia, Lachnospiraceae NK3A20 group, Triticum aestivum_bread_wheat, Sanguibacter, Roseburia. Mycobacterium, Ruminococcus 2, Anaerotruncus, Alkanindiges, Glycomyces, Shuttleworthia, Finegoldia, Chitinophaga, Gaiella, Halomonas, Rubrobacter, Planomicrobium. Nocardia, Microvirga, Peptoniphilus, RB41, Phyllobacterium, Ramlibacter, Promicromonospora, Flavobacterium, Steroidobacter, Propionivibrio, freshwater_sediment_metagenome, Flavitalea, Amycolatopsis, Leuconostoc. Ruminococcaceae NK4A214 group, Escherichia-Shigella, Anaerococcus. Ohtaekwangia, Christensenellaceae R-7 group, Variibacter, Herbaspirillum, Ensifer, Vagococcus, Chroococcidiopsis, Roseiflexus, Luteimonas, Blastocatella, Gardnerella, Desemzia, Ralstonia, Pediococcus, Brachybacterium, Blastococcus, Facklamia, Roseiarcus, Deinococcus, Oceanobacillus, Catenulispora, Kribbella, Brochothrix, Agromyces, Methylosinus, Subdoligranulum, Sphingobacterium, Acetitomaculum, Polaromonas, Brachymonas, Iamia, Aerococcus, Chryseomicrobium, Acidothermus, Bryobacter, Candidatus_Solibacter, Nakamurella, Reyranella, Dactylosporangium, Mesorhizobium, Aeromicrobium, Weissella, Moryella, Pedomicrobium, Kocuria, Syntrophus, Shinella* |
|  | H | *Fluviicola, Aridibacter, Flavisolibacter, Anaeroglobus, Hymenobacter, Ezakiella, Atopostipes, Defluviicoccus, Caulobacter, Bacteroides, Blautia, Holdemanella, Tessaracoccus, Dolosigranulum, Prevotella 9, Bosea, Bulleidia, Clostridium_sensu_stricto_1, Ruminococcaceae_UCG-013* |
| Saliva | T | *Hyphomicrobium, Cellvibrio, Pseudohongiella, Candidatus_Stoquefichus, Taibaiella, Nordella, Fluviicola, Cnuella, Sphingopyxis, Vulcaniibacterium, Humulus_lupulus_var._lupulus, Nocardioides, Terrimonas, Pseudonocardia, Lachnospiraceae NK3A20 group, Flavisolibacter, Firmicutes oral clone FM046, Undibacterium, Nitrosomonas, Nonomuraea, Ruminococcus 2, Cupriavidus, Schlegelella, Gemmatimonas, Micrococcus, Rhizomicrobium, Sphingobium, Saccharothrix, Ezakiella, Eubacterium_saphenum_group, Glycomyces, Mucilaginibacter, Mycoplasma, Finegoldia, Chitinophaga, Gaiella, Microvirga, Adhaeribacter, Peptoniphilus, RB41, Luedemannella, Ramlibacter, Promicromonospora, Flavobacterium, CL500-29 marine group, Flavitalea, Amycolatopsis, Chryseobacterium, Opitutus, Ruminococcaceae NK4A214 group, Escherichia-Shigella, Caulobacter, Anaerococcus, Niastella, Ohtaekwangia. Candidatus_Amoebophilus, Blautia, Roseiflexus, Lactococcus, Gardnerella, Gordonia, Pediococcus, Hydrogenophaga, Azospira, Collinsella, Ochrobactrum, Blastococcus, Roseiarcus, Rubellimicrobium, Deinococcus, Parasegetibacter, Variovorax, Kribbella, Azospirillum, Methylosinus, Altererythrobacter, Acetitomaculum, Methylotenera, Cryptobacterium, Brachymonas, Pontibacter, Iamia, Arenimonas, Aerococcus, Meiothermus, Panacagrimonas, Telmatospirillum, Sorangium, Paucimonas, Rudaea, Nakamurella, Reyranella, Dactylosporangium, Enterococcus, Aeromicrobium, Weissella, Moryella, Pelomonas, Jatrophihabitans, Pedomicrobium, Limnobacter, Rhodobacter, Shinella* |
|  | H | *Edaphobacter, Actinospica, Hymenobacter, Aquabacterium, Nocardia, Shewanella, Psychrobacter, Corynebacterium 1, Vagococcus, Slackia, Methyloversatilis, Clostridium_sensu_stricto_1, Helcococcus, Kocuria* |
| Supragingival Plaque | T | *Dyella, Streptomyces, Hyphomicrobium, Taibaiella, Fluviicola, Sphingopyxis, Pseudarthrobacter, Schrenkiella_parvula, Nocardioides, Terrimonas, Pedobacter, Pseudonocardia, Lachnospiraceae NK3A20 group, Triticum_aestivum_bread_wheat, Edaphobacter, Actinospica, Roseburia, Nonomuraea, Mycobacterium, Ruminococcus, Pseudoxanthomonas, Rhizomicrobium, Ezakiella, Mucilaginibacter, Aquabacterium, Chitinophaga, Gaiella, Eubacterium_hallii_group, Peptoniphilus, Sediminibacterium, RB41, Luedemannella, Ramlibacter, Promicromonospora, Flavobacterium, Ilumatobacter, CL500-29 marine group, Devosia, Flavitalea, Ruminococcaceae NK4A214 group, Escherichia-Shigella, Anaerococcus, Christensenellaceae R-7 group, Variibacter, Ensifer, Romboutsia, Thermosporothrix, Holdemanella, Ruminococcaceae UCG-002, Lysobacter, Gardnerella, Ruminococcaceae UCG-005, Gordonia, Rhodoplanes, Ralstonia, Pediococcus, Hydrogenophaga, Ochrobactrum, Rhizobium, Roseiarcus, Bosea, Lachnospiraceae XPB1014 group, Parasegetibacter, Variovorax, Kribbella, Azospirillum, Methylosinus, Subdoligranulum, Acetitomaculum, Dyadobacter, Brachymonas, Iamia, Arenimonas, Aerococcus, Acidothermus, Bryobacter, Ruminococcaceae UCG-013, Clostridium sensu stricto 1, Candidatus_Solibacter, Telmatospirillum, Family XIII AD3011 group, Candidatus_Xiphinematobacter, Paucimonas, Nakamurella, Reyranella, Granulicella, Mesorhizobium, Candidatus_Soleaferrea, Kocuria, Shinella* |
|  | H | *Segetibacter, Serratia, Stenotrophomonas, Geodermatophilus, Flavisolibacter, Undibacterium, Alkanindiges, Shewanella, Psychrobacter, Skermanella, Slackia, Dolosigranulum, Novosphingobium, Methylophilus, Brochothrix, Methylobacterium, Chryseomicrobium, Helcococcus, Limnobacter* |
